# Early deviation from normal structural connectivity: A novel intrinsic severity score for mild TBI

**DOI:** 10.1212/WNL.0000000000008902

**Published:** 2020-03-10

**Authors:** Peter Neal Taylor, Nádia Moreira da Silva, Andrew Blamire, Yujiang Wang, Rob Forsyth

**Affiliations:** From the Interdisciplinary Complex Systems Group, School of Computing (P.N.T., N.M.d.S., Y.W.), Institute of Neuroscience, Faculty of Medical Sciences (P.N.T., Y.W., R.F.), and Institute of Cellular Medicine & Newcastle MR Centre (A.B.), Newcastle University, Newcastle Upon Tyne; and Institute of Neurology (P.N.T., Y.W.), University College London, UK.

## Abstract

**Objective:**

Studies of outcome after traumatic brain injury (TBI) are hampered by the lack of robust injury severity measures that can accommodate spatial-anatomical and mechanistic heterogeneity. In this study we introduce a Mahalanobis distance measure (*M*) as an intrinsic injury severity measure that combines in a single score the many ways a given injured brain's connectivity can vary from that of healthy controls. Our objective is to test the hypotheses that *M* is superior to univariate measures in (1) discriminating patients and controls and (2) correlating with cognitive assessment.

**Methods:**

Sixty-five participants (34 with mild TBI, 31 controls) underwent diffusion tensor MRI and extensive neuropsychological testing. Structural connectivity was inferred for all participants for 22 major white matter connections. Twenty-two univariate measures (1 per connection) and 1 multivariate measure (*M*), capturing and summarizing all connectivity change in a single score, were computed.

**Results:**

Our multivariate measure (*M*) was able to better discriminate between patients and controls (area under the curve 0.81) than any individual univariate measure. *M* significantly correlated with cognitive outcome (Spearman ρ = 0.31; *p* < 0.05). No univariate measure showed significant correlation after correction for multiple comparisons.

**Conclusions:**

Heterogeneity in the severity and distribution of injuries after TBI has traditionally complicated the understanding of outcomes after TBI. Our approach provides a single, continuous variable that can fully capture individual heterogeneity. *M*'s ability to distinguish even mildly injured patients from controls and its correlation with cognitive assessment suggest utility as an imaging-based marker of intrinsic injury severity.

Traumatic brain injury (TBI) is a major public health issue in both developed and developing countries. Attempts to identify superior treatment strategies through the use of between-group randomized controlled trials have largely failed as it is difficult to identify treatment effects against the “noise” of an extremely heterogeneous condition.^1^ More recently, researchers have adopted comparative effectiveness research (CER) methodologies both in relation to intensive care unit (ICU) and post-ICU treatments.^2^ Such methods require the definition of severity-adjusted expectations of outcome so that differences between observed and expected severity-adjusted outcome can be related to treatment received.

Robust methods for the objective quantification of TBI severity against which to regress observed recovery are surprisingly lacking. The limitations of clinical measures such as the initial Glasgow Coma Scale score are well-recognized.^3^ There have been extensive efforts to quantify secondary ischemic insult due to raised intracranial and reduced cerebral perfusion pressure but again these explain a small proportion of the variance in late outcome, in part because many pathologies other than the ischemic insults reflected in these hemodynamic measures contribute to late outcome.^4^ In particular, traumatic axonal injury (TAI), a major factor in the neuropathology of TBI and an important determinant of late outcome, does not arise from ischemic processes and is poorly reflected in such data. Pragmatically, various time to attainment of functional recovery milestone measures are used as proxies of injury severity. Examples include the duration of posttraumatic amnesia and time to follow commands. However, the use of early rate of recovery measures to predict subsequent recovery is unsatisfactorily tautological.

Objective, intrinsic measures of TBI severity would be of considerable value in TBI CER, and imaging-based approaches have an obvious appeal. CT-based injury severity scales have been devised^5^ but these are (1) nonquantitative and (2) less useful in mild to moderate injury, which is by far the most common subtype. MRI has been shown to be more sensitive to structural injury and fractional anisotropy (FA) has been used frequently to quantify injury that impairs white matter axonal integrity such as TAI. In this study, we develop a continuous severity score reflecting the extent to which an individual's FA data are an overall outlier from control population data, and test its association with cognitive assessment.

## Methods

### Neuroimaging

The imaging data are reported in a previous study.^6^ Thirty-four patients were scanned using a 3T MRI scanner within a mean of 5.5 days of mild injury (range 1–14 days, SD 2.7 days). The scanning sequence included T1-weighted and diffusion tensor protocols described previously.^6^ Thirty-one controls matched for age, sex, and education level were also imaged. All participants gave written informed consent and the study was approved by the local ethics committee.

FA values were derived along major white matter tract bundles using the following procedure. First, native space FA values were derived using the DTIFit tool from FSL after eddy correction using the eddy_correct tool. Second, native space FA maps were warped to a standard space using the T1-weighted MRI to improve the registration. This second step was performed using DSI studio with constrained diffeomorphic registration. Third, using DSI studio, we overlaid 22 manually identified white matter association tract bundles^7^ to derive a mean FA for each bundle in each participant. This was performed by generating a connectometry database comprising all participants’ standard space FA maps, then selecting the 22 tracts of interest in the tractography step in DSI studio. For each tract bundle, the mean FA was exported.^8^ We restricted ourselves to association tract bundles because of their hypothesized relation with cognitive function (see Cognitive functioning section and references 9 and 10) and to ensure the number of tracts analyzed is less than the number of control participants. Age as a covariate was regressed out using the fitlm method in MATLAB, and the residuals (FA^r^) were used to compute univariable and multivariate distances.

### Distance measure to capture injury heterogeneity

**Figure 1:**
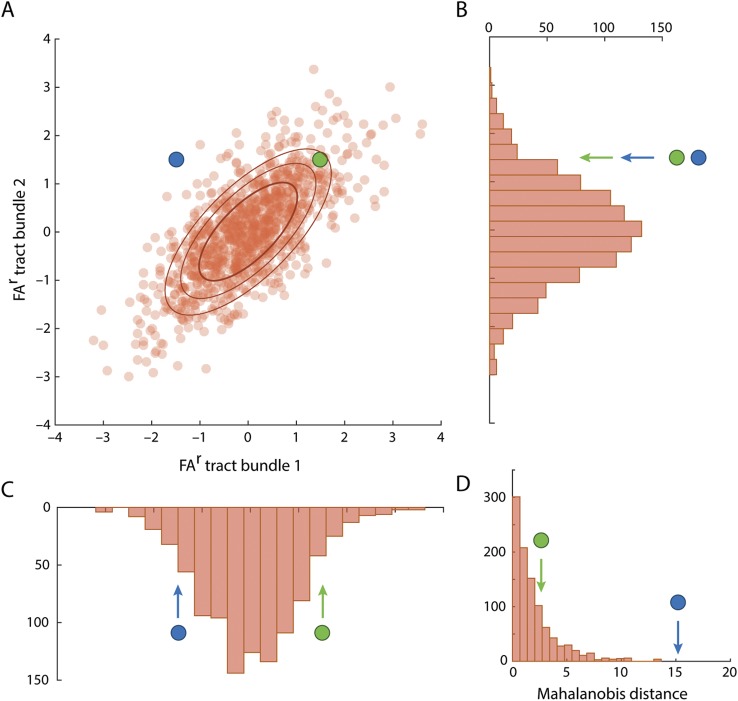
Schematic illustration of the Mahalanobis distance concept Readers will be familiar with the description of normally distributed continuous data (e.g., height) for an individual as a *z* score. The distance of that individual's height from the population mean is expressed in SD units. Note that this is a probability distance: a quantification of how unusual the individual is as a member of the population. For normally distributed data, a *z* score of 2 corresponds to a 1-tailed probability of 2.2% of being at least that far from the mean. Extending to the 2-dimensional case, consider a population height and weight dataset. Height and weight are positively correlated and a height-weight scatterplot will resemble (A). The probability distribution that for height alone was a Gaussian bell curve is now represented by contour lines. Individuals can now be outliers for various combinations of height and weight, but the extent to which an individual is an outlier (the probability distance measure) can still be represented by a single number reflecting the contour the individual is on. Different height-weight combinations can have the same probability distance. Because height and weight are correlated, the contours are ellipses rather than circles: separation from the population centroid in a direction perpendicular to the long axis of the ellipses is more unusual than separation by the same distance along the long axis. For a 3-dimensional dataset (height, weight, and shoe size), the probability distribution contours are now nested ellipsoids, but the probability distance measure is still a single number. In multidimensional space, this distance measure is known as the Mahalanobis distance (*M*).^21^ Here we are using *M* to capture the probability distance of an individual's post traumatic brain injury (TBI) MRI fractional anisotropy (FA) data from those of controls. Although *M* is unidimensional, it captures distance in multivariate space (here, the 22-dimensional FA^r^ dataset). Despite anatomical heterogeneity of injuries, we can identify equal levels of distance from the control dataset. (A) Schematic orange scatter points illustrate an example covariance between FA^r^ in 2 tracts in a simulated healthy population (each point represents a control participant). Concentric ellipses illustrate the density of the scatter points, and are equidistance lines for the Mahalanobis distance (*M* = 1, 2, 3). Blue and green points represent 2 individuals. In univariate analyses (plots B and C), the green and blue participants both have FA^r^ values within 2 SDs of the mean for both tracts. However, multivariate analysis that accounts for the covariance between the FA^r^s of tracts 1 and 2 (D) shows that the blue participant (*M* = 15.20) is much further away from the control distribution than the green participant (*M* = 2.64). The blue individual's combination of low FA^r^ in tract 1 and high FA^r^ in tract 2 is particularly unusual (compare an individual who is unusually short given his weight). The increased distance is also visually apparent in (A), where the blue participant is further from the control distribution in the 2-dimensional space. Thus, one might hypothesize that the blue participant is a participant with TBI since she or he is far from the control distribution.

The Mahalanobis distance is the multivariate generalization of the well-known *z* score that incorporates covariance between the variables. For further explanation, see the legend to figure 1. It is formally defined as follows:
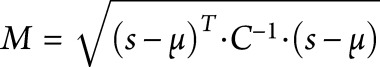
where *s* = [FA^r^_1_, FA^r^_2_… FA^r^_22_] is the vector of FA^r^ observations in each tract bundle in a single participant; μ is the vector of mean FA^r^ of each tract bundle in a healthy control population; and *C* is the covariance matrix between tract bundles across that control population.

Mindful of the large number of tract bundles (22) relative to the number of controls (31) and patients (34), we used conservative approximations of *C* in place of the conventional Pearson correlation. Shrinkage estimators^11^ remove potentially spurious correlations in small datasets. To ensure robustness, we used a subset of 25 randomly selected controls to compute the covariance and permuted this 1,000 times. We estimated the univariate and multivariate distances of each patient to each of the 1,000 control distributions and report the median values. We computed distances of controls to the remaining controls using the same permutation-based approach, with the exception that the control being computed was held out from the construction of *C*. As a univariate measure for comparison we calculated the analogue of the |*z* score| using the same permutation-based approach. These conservative estimations of *Z* and *M* are in all other respects directly analogous to those derived from standard Pearson correlation estimation in larger samples. Conceptually, our approach is illustrated in figure 1.

### Cognitive functioning

Patients and controls underwent a battery of standardized neuropsychological tests sensitive to cognitive impairments in attention, memory, executive functions, and semantic knowledge within 14 days of scanning. We restricted our analysis to the following tests: Speed of Information Processing, Delis-Kaplan Executive Function System Color-Word Interference Test, Category Fluency, and List Learning.

Principal component analysis (PCA) was used to create a combined score representing an overall degree of neurocognitive function. Since PCA is unsuitable for highly skewed data,^12^ a Box-Cox transformation was applied to cognitive scores with *z* skewness and *z* kurtosis higher than 1.96.^13^ As a summary cognitive score, the first principal component (explaining 63% of the variance) was used as the dependent variable in analyses.

### Data availability

Data and code to reproduce the figures is avaliable at 10.5281/zenodo.3593087.

## Results

### Univariate and multivariate distances to discriminate between patients and controls

**Figure 2:**
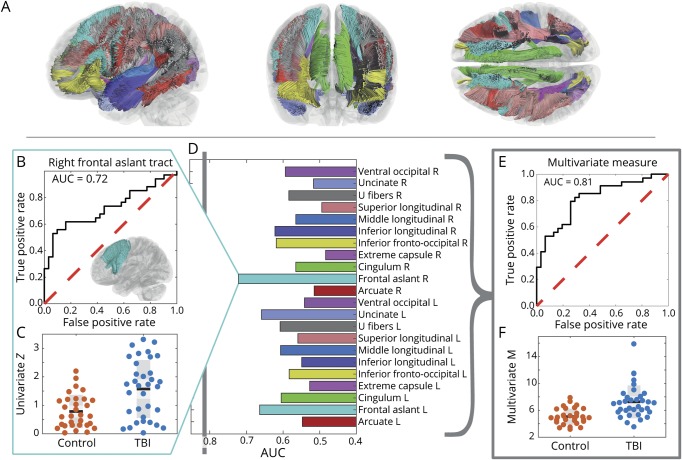
Multivariate *M* is superior to univariate *Z* in discriminating injured patients from controls (A) Major tracts used in this study: colors correspond to the tracts identified in panel D. (B) Receiver operator characteristic (ROC) curve for the best performing univariate *Z* measure: that for the right frontal aslant tract. (C) Data underlying B and D. ROC area under the curve (AUC) values using univariate *Z* (effectively, bootstrapped *z*) scores for each individual tract. (E) ROC-AUC curve for the multivariate *M* distance measure. (F) Data underlying E. TBI = traumatic brain injury.

We investigated the ability of univariate *Z* distance to discriminate patient and control groups for each of the 22 tract bundles individually (shown in figure 2A). The right frontal aslant tract had the best discriminatory ability with an area under the receiver operator characteristic curve (AUC) of 0.72 (figure 2, B–D). However, considering all tracts and their covariance using the multivariate *M* distance has a superior discriminating ability, with an AUC of 0.81 (figure 2, E and F).

### Multivariate distance correlates with functional performance in patients

**Figure 3:**
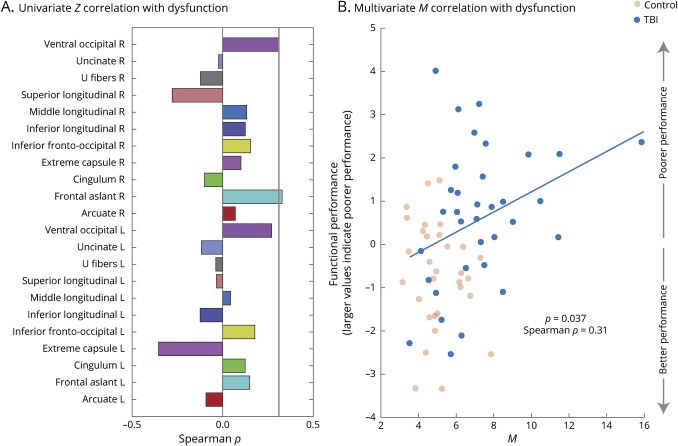
Only multivariate *M* distance is significantly correlated with cognitive performance (A) Correlation between the univariate *Z* measure for each tract for each injured individual and the summary cognitive performance score (first component of the principal component analysis of the multiple cognitive function tests: high scores imply poorer performance). No univariate correlation is significant after correction for multiple comparisons. (B) Scatterplot shows the significant (*p* = 0.037) correlation between multivariate distance measure *M* and functional performance in patients. Line of best fit uses bisquare regression robust to outliers. TBI = traumatic brain injury.

Since neurocognitive functioning integrates information from multiple brain areas, we hypothesized that multivariate *M* distances would be positively correlated with poorer functional performance in patients. Figure 3 shows the Spearman correlation between the first component of the PCA-derived summary neurocognitive function measure and the univariate *Z* and multivariate *M* distances. The multivariate *M* distance is significantly positively correlated with neurocognitive function (*p* < 0.05, ρ = 0.31). However, of all univariate distances, only one has a higher positive correlation that is not significant after correction for multiple comparisons (false discovery rate-corrected *p* > 0.05) (*p* values associated with the *M* distance measure are not corrected for multiple comparisons as only a single comparison is performed, unlike for the multiple individual univariate measures).

## Discussion

In this study, we demonstrate the feasibility of deriving a single continuous intrinsic injury severity variable capturing the distance of an individual's white matter integrity (FA), considered in its entirety, from controls. The greater sensitivity to deviation from control data comes from incorporation of knowledge of the covariance structure of the control population connectivity dataset. We demonstrate the multivariate *M* measure's superiority over all univariate measures in discriminating patients from controls and its association with neurocognitive function after TBI, as measured by a consolidated measure of neurocognitive function.

There are a number of technical statistical advantages to the measure, including (1) multiple comparison correction is not required as only 1 test is performed and (2) independence from measurement noise assuming the latter is independent, normally distributed, and noncovarying. As a continuous, scalar variable, *M* is well-suited to use as an intrinsic severity measure in TBI outcome studies and it avoids tautologous measures of injury severity such as PTA that use an early time-to-recovery milestone measure to predict rates of attainment of later stages of clinical recovery. An important principle of this approach is that it downplays the significance of the anatomical location of lesions: it is thus likely to be more applicable and useful in spatially diffuse injuries such as those following TBI.^14^ Similarly, the approach could be applied in other heterogeneous conditions such as multiple sclerosis.

Limitations of our study include the relatively small sample size (the use of shrinkage estimators to derive conservative estimates for *C* mitigates this here but would not be required in studies with larger participant numbers), the limited number of tracts, and the timing of the imaging. As the scans were done very soon after injury, there is some uncertainty regarding the exact pathophysiology an increased FA-based *M* distance reflects. This may be a particular issue for *M* distances derived from early diffusion-weighted imaging measures such as FA, which may require consistent timing postinjury. FA changes can be detected within hours after TBI and evolve over months.^15^ Animal models suggest hyperacute FA changes may reflect acute edema.^16,17^ Postacute diffusion tensor imaging changes appear more stable and reflect gliosis.^18,19^ However, the general *M* distance approach could be applied to magnetic resonance modalities reflecting other pathophysiology that may be more stable over time (such as susceptibility-weighted imaging).^18,20^ To achieve use in a clinical setting as a diagnostic tool, control cohorts would likely be required to build *C*, similarly matched in demographics to the patient, and ideally acquired on the same scanner.

An obvious extension of this work would be to examine longitudinal change in *M*, and movement through the high-dimensional FA space, during recovery. We envisage this may also permit the development of personalized of rehabilitation strategies aimed at optimizing individual trajectories through this high dimensional FA “recovery space.”

## Glossary

AUC: area under the receiver operator characteristic curve
CER: comparative effectiveness research
FA: fractional anisotropy
ICU: intensive care unit
PCA: principal component analysis
TAI: traumatic axonal injury
TBI: traumatic brain injury
